# The effects of the Korean reference value on the prevalence of osteoporosis and the prediction of fracture risk

**DOI:** 10.1186/s12891-015-0523-4

**Published:** 2015-03-27

**Authors:** Sungwha Lee, Moon-Gi Choi, Jaemyung Yu, Ohk-Hyun Ryu, Hyung Joon Yoo, Sung-Hee Ihm, Doo-Man Kim, Eun-Gyung Hong, Kyutae Park, Myungjin Choi, Hyunhee Choi

**Affiliations:** Department of Internal Medicine, Chuncheon Sacred Heart Hospital, Hallym Medical University, Chuncheon Sacred Heart Hospital, 77, Sakju-ro, Chuncheon-si, Gangwon-do South Korea; Department of Internal Medicine, Gangnam Sacred Heart Hospital, Hallym Medical University, 1, Singil-ro, Seoul, 150-950 Yeongdeungpo-gu South Korea; Department of Internal Medicine, Hallym University Sacred Heart Hospital, 22, Gwanpyeong-ro 170 beon-gil, Dongan-gu, Anyang-si, 431-796 Gyeonggi-do South Korea; Department of Internal Medicine, Kangdong Sacred Heart Hospital, Hallym Medical University, 150, Seongan-ro, Seoul, 134-701 Gangdong-gu South Korea; Department of Internal Medicine, Dongtan Sacred Heart Hospital, Hallym Medical University, 7, Keunjaebong-gil, Hwaseong-si, 445-907 Gyeonggi-do South Korea

**Keywords:** Bone mineral density, Fracture risk, FRAX, Korean reference value, Osteopenia, Osteoporosis, Prediction of fracture risk, Prevalence, Reference, T-score

## Abstract

**Background:**

Since the reference value is the core factor of the T-score calculation, it has a significant impact on the prevalence of osteoporosis. The purpose of this study was to determine the effects of using the Korean reference value on the prevalence of osteoporosis and on the prediction of fracture risk.

**Methods:**

We used femoral neck bone mineral density (BMD) data from the Korea National Health and Nutrition Examination Survey (KNHANES) 2008–2011. The Korean reference was identified by the mean and standard deviation of men and women aged 20–29 years. We compared the prevalence and the fracture risk assessment tool (FRAX™) probability obtained from the Korean reference and the NHANES III reference.

**Results:**

In men, the prevalence of osteoporosis increased when using the Korean men’s reference, and the difference increased up to 9% for those in their 80s. In women, the prevalence increased when using the NHANES III reference, and the difference increased up to 17% for those in their 80s. The reference value also affected the fracture risk probability, and the difference from changing the reference value increased in women and in subjects with more clinical fracture risk factors. In major osteoporotic fractures, the difference of the risk probability was up to 6% in women aged 70–79 years with two clinical risk factors. For femoral neck fractures, the difference was up to 7% in women aged 50–59 years with two clinical risk factors.

**Conclusions:**

We confirmed that the reference value had significant effects on the prevalence of osteoporosis and on the fracture risk probability. The KNHANES 2008–2011 BMD data reflected the characteristics of the Korean BMD status well with regard to data size and study design; therefore, these data can be used as reference values.

## Background

The diagnostic criteria for osteoporosis are based on the T-score, which is calculated from the mean bone mineral density (BMD), and its standard deviation of the reference from the young adult group in the World Health Organization’s (WHO) consensus of 1993 [1]. Because the T-score depends on the value of a reference group, the definition of the reference group has been controversial [2-8]. While one group suggested using the universal reference BMD of 20–29 year-old non-Hispanic white women from the National Health and Nutrition Examination Survey (NHANES) III, another group suggested using the regional reference BMD data of a region- and race-specific young adult group. Meanwhile, the main guidelines suggest the former, and this is based on several reasons [9-12]. First, the prevalence of each group of osteoporosis is significantly different, but it is small when compared with the difference in fracture incidence by region. Therefore, the benefit of using local reference BMD data in terms of predicting fracture is not clear [12,13]. Second, it is realistic to use the NHANES III BMD data, because it is large and well sampled enough in areas where there is no equivalent BMD data.

The aforementioned reasons may be valid for using a universal reference in an area where osteoporosis treatment is based on the assessment of fracture risk. If we use the fracture risk assessment tool (FRAX™), the BMD value itself is used rather than the T-score. Therefore, the decision for medical treatment will receive less influence from the T-score and the reference value. However, in many regions, the use of medical prediction tools such as the FRAX™ is limited, and osteoporosis diagnosis and treatment is based on the T-score [14]. Thus, in such areas, the T-score and related reference BMD data still have great influence on the diagnosis and treatment of osteoporosis and the standard for reimbursement. Additionally, the drug holiday, which is based on the risk assessment according to the T-score after 3–5 years of bisphosphonate therapy, is suggested [15-19]. The reference BMD value also has a significant impact in this case.

The first aim of this study was to estimate the effect of changing the reference value (obtained from the Korean BMD data and white women BMD data from the NHANES III) on the prevalence of osteoporosis by sex and age group. The second aim was to evaluate the effects of the change of reference value on the fracture risk probability by comparing the FRAX™ 10-year risk probability obtained from each T-score. In Korea, there is no clear definition for the reference group; thus, our study may be used as important material for determining how to choose the reference value for diagnosing osteoporosis in Korea.

## Methods

### Subjects

We used the BMD data from the Korea National Health and Nutrition Examination Survey (KNHANES; 2008–2011). This survey is administered nationwide and has been conducted since 1995 in South Korea by the Korea Centers for Disease Control and Prevention. The KNHANES from 2008–2009 was administered to 4,600 households from 200 primary sampling units, while the KNHANES from 2010–2011 was administered to 3,800 households from 192 primary sampling units each year. First, the primary units were selected by the administrative regions; one study unit was extracted from each area after considering the type of house. Second, 20 households from each study unit were extracted using a systematic sampling method, and these households were the basic units in the survey. By using the rolling survey sampling, a sample from each year was representative of the population of that respective year.

The KNHANES was composed of a health interview survey, nutritional survey, and a health examination survey. The health interview and health examination surveys were administered in a mobile examination center, and the nutritional survey was administered by direct visit and interview. The health interview survey was administered via a direct interview when the subjects visited the mobile center; however, alcohol use and current smoking status were surveyed by using a self-reporting questionnaire. The health examination survey was analyzed by using measurements, observation, and laboratory sampling.

The subjects were men and women who received the test for BMD during the KNHANES from 2008–2011 [20,21]. There was no significant difference between subjects who did not receive a BMD scan and those who did; thus, the risk of selection bias was very low. The subjects used for investigating the Korean reference value were 20–29-year-old males and females. The analysis comparing the prevalence and fracture risk probability was conducted to target men and postmenopausal women >50 years. Menopause was determined by the subjects’ responses to the following survey items: (1) those who answered natural menopause or menopause-related procedure to the question, why do you not menstruate?; (2) when the subjects’ period since the last menstruation was >1 year; and (3) those who answered yes or bilateral ovariectomy to menopause status. Women who satisfied all three conditions were considered postmenopausal. Postmenopausal women from 50–64 years and women >65 years were included in the analysis of the prevalence of osteoporosis and fracture risk probability. The BMD data from the NHANES III were used to compare the BMD of white women from the United States NHANES III to the value of men and women from the KNHANES [22]. The content and methodology of the KNHANES was approved by the institutional review board of the Korea Centers for Disease Control and Prevention, and all the participants signed informed consent.

### Measurement of bone mineral density, the reference value, and diagnosis for osteoporosis

The BMD test was performed by a trained radiographer in a mobile examination center using dual-energy radiography absorptiometry (DXA; DISCOVERY-W fan-beam densitometer; Hologic, Bedford, MA, USA) of the lumbar spine (L1–L4) and proximal hip (femoral neck and total femur). Hologic Discovery software, version 3.1 was used for that device. The accuracy was investigated by performing double scans on 30 randomly selected subjects, and the accuracy was maintained within the range of 0.73–1.07% for the lumbar spine, 1.20–2.14% for the femoral neck, and 0.71–1.18% for the total hip. Daily calibration was performed by a phantom served by the company.

In this study, we analyzed the BMD of the femoral neck. The reference value for Koreans was set to the mean BMD and its standard deviation of men and women aged 20–29 years. We used the mean BMD and standard deviation (SD) (0.858 ± 0.120 g/cm^2^) published in a previous study as the NHANES III reference [23]. Osteoporosis, osteopenia, and normal were defined using the following T-scores according to the WHO criteria: ≤ − 2.5, −1.0 to −2.5, and −1.0, respectively.

### Estimation of the fracture probability that corresponds to each reference

The 10-year fracture risk was calculated by the Korea FRAX™ version to assess how the change in the reference affects the relationship between BMD and fracture risk prediction [24]. The BMD that corresponds to each T-score −1.5, −2, −2.5, −3.0, −3.5, and −4.0) will vary depending on the reference value. For example, a T-score of −2.5 corresponded to 0.5128 g/cm^2^ when using a Korean women reference and to 0.5553 g/cm^2^ when using a NHANES III reference. When these different BMDs were used in FRAX™, the fracture risk probability was calculated as 6% and 6.8%, respectively. For weight and height, the mean values of each age and sex group were used. The difference of fracture risk probability by the references was analyzed by age group and by the number of clinical risk factors for fracture. To simplify the analysis, only a history of fracture and a family history of hip fracture in a parent of two estimates were analyzed.

### Statistical analysis

Statistical analyses were performed using SPSS for Windows, version 21.0 (SPSS Inc., Chicago, IL, USA). Continuous variables were described in the form of mean ± SD; the prevalence was presented as a percentage (%). Firstly, the subjects were partitioned into 10-year age groups, and the mean and SD of BMD of each age group was calculated. The mean BMDs did not satisfy the assumption of equal variance, so the Welch’s test and Tamhane’s T2 post-hoc analysis were used to test the significance of the difference between the mean BMD by age group. To test whether there was a significant difference between the prevalence calculated by the Korean reference and by the NHANES III reference, the McNemar–Bowker test and Cohen’s kappa coefficient were used to evaluate the degree of diagnostic agreement.

## Results

### Mean and standard deviation of bone mineral density by age groups

**Figure 1:**
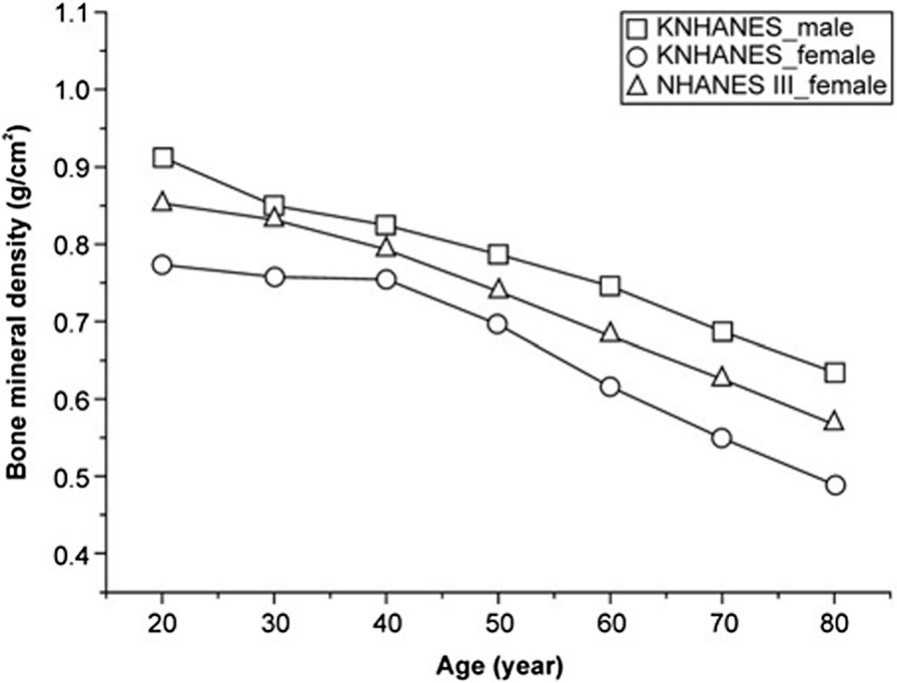
**The mean and standard deviation of bone mineral density (BMD) by age group from the Korea National Health and Nutrition Examination Survey (KNHANES) 2008–2011 and NHANES III.** The mean BMD of KNHANES men, women, and NHANES III white women were compared according to age group. The square, triangle, and circle represent the mean BMD of the KNHANES males, NHANES III white women, and KNHANES females, respectively.

The basic characteristics of the subjects are summarized in Table 1. The mean BMD, height, and weight decreased as the subjects’ age increased. In Table 2, the mean and standard deviation of BMD of each age group was compared with the Korean BMD data and the NHANES III BMD data. The mean BMD of Korean men, women, and women in the NHANES III decreased with increasing age. There was a significant difference between the mean BMD of the 20–29-year-old age group and that of another age group in the KNHANES 2008–2011 (Table 3). However, in the NHANES III data, there was no significant difference between those in their 20s and 30s. Table 3 and Figure 1 shows the decrease in the mean BMD with increasing age by the reference groups. The mean BMD was identified from highest to lowest across all age groups as follows: Korean men > NHANES III women > Korean women.

**Table 1:** **Baseline characteristics of the subjects**

**Male**	**Age**	***n***	**BMD (g/cm** ^**2**^ **)**	**Height (cm)**	**Weight (kg)**
	**50–59**	1,448	0.7879 ± 0.1118	168.36 ± 5.70	68.64 ± 9.26
	**60–69**	1,410	0.7453 ± 0.1125	166.23 ± 5.66	65.78 ± 9.25
	**70–79**	958	0.6864 ± 0.1103	164.56 ± 5.65	62.08 ± 9.52
	**80–89**	182	0.6329 ± 0.1176	162.36 ± 5.60	58.38 ± 8.90
**Female**	**Age**	***n***	**BMD (g/cm** ^**2**^ **)**	**Height (cm)**	**Weight (kg)**
	**50–59**	1,395	0.6832 ± 0.0960	155.47 ± 5.21	58.39 ± 8.02
	**60–69**	1,697	0.6156 ± 0.0870	153.40 ± 5.21	57.71 ± 8.17
	**70–79**	1,282	0.5486 ± 0.0876	150.04 ± 5.55	54.36 ± 8.92
	**80–89**	304	0.4875 ± 0.0831	147.10 ± 5.49	49.63 ± 8.72

BMD, bone mineral density.

**Table 2:** **The reference values (gm/cm** ^**2**^ **) for femoral neck fractures**

	**Mean**	**SD**	Cut-off BMD* (gm/cm ^**2**^ **)**
**KNHANES 2008–2011 Male**	0.9123	0.1336	0.5783
**NHANES III Female**	0.8525	0.1189	0.5553
**KNHANES 2008–2011 Female**	0.7740	0.1045	0.5128

SD, standard deviation; BMD, bone mineral density; KNHANES, Korea National Health and Nutrition Examination Survey.

*Cut-off BMD: BMD corresponding to a T-score of −2.5.

**Table 3:** **The mean bone mineral density (g/cm** ^**2**^ **) by age group**

**Age**	**Male KNHANES 2008–2011**				**Female KNHANES 2008–2011**				**NHANES III**			
	***n***	**Mean**	**SD**	**T** ^*^	***n***	**Mean**	**SD**	**T** ^*^	***n***	**Mean**	**SD**	**T** ^*^
20–29	976	0.9123	0.1336	a	1,236	0.7740	0.1045	a	409	0.8525	0.1189	a
30–39	1,569	0.8507	0.1195	b	2,098	0.7587	0.1030	b	518	0.8310	0.1210	a
40–49	1,591	0.8254	0.1160	c	2,071	0.7572	0.1043	b	444	0.7934	0.1273	b
50–59	1,449	0.7879	0.1118	d	1,996	0.6976	0.1011	c	450	0.7401	0.1196	c
60–69	1,412	0.7453	0.1125	e	1,778	0.6159	0.0881	d	454	0.6820	0.1206	d
70–79	962	0.6864	0.1103	f	1,283	0.5486	0.0876	e	556	0.6245	0.1088	e
80–89	183	0.6329	0.1176	g	306	0.3982	0.0809	f	420	0.5694	0.1057	f

SD, standard deviation; KNHANES, Korea National Health and Nutrition Examination Survey.

Welch test: p < 0.001.

^*^Post-hoc analysis (Tamhane’s T2); the letters represent the significant difference by age group.

### Agreement of diagnosis by each reference

If an agreement was found in the diagnosis of osteoporosis based on each reference value, it was represented by the sex and age group in Tables 4 and 5. For men, the results of the diagnosis were calculated using the Korean reference and the NHANES III women reference, which were fairly consistent. It was estimated by age groups as follows: 50s: *k* = 0.668, concordance 83.3%; 60s: *k* = 0.661, concordance 81.1%; 70s: *k* = 0.654, concordance 81.4%; and 80s: *k* = 0.633, concordance 78.1%. The concordance rate was slightly decreased as age increased. Despite a high concordance, there were statistically significant differences between the diagnosis and each reference (McNemar-Bowker test, p < 0.001). A significant difference was also found in women; however, the degree of agreement was lower than that of men. It was estimated by age groups as follows: 50s: *k* = 0.457, concordance 69.5%; 60s: *k* = 0.446, concordance 71.1%; 70s: *k* = 0.553, concordance 75%; and 80s: *k* = 0.565, concordance 82%. While the difference in the diagnosis was increased in aging men, the difference in the diagnosis in women was decreased with an increasing age. However, with regard to osteoporosis, the difference in the prevalence of osteoporosis was increased in both sexes, except in women aged >80 years.

**Table 4:** **The change in diagnosis by the selected reference for males**

	***Cohen’s k***		**% of agreement**						**Prevalence difference**
**50s**	***k*** **= 0.668**		**Agreement = 83.3%**						**1%**
			**NHANES diagnosis**						
**KNHANES diagnosis**			Normal		Osteopenia		Osteoporosis		
	*n*	%	*n*	%	*n*	%	*n*	%	
Normal	744	51.40	744	51.4	0	0	0	0	
Osteopenia	685	47.30	230	15.9	455	31.4	0	0	
Osteoporosis	19	1.30	0	0	11	0.8	8	0.5	
Total	1,448	100.00	974	67.3	466	32.2	8	0.5	
**60s**	***k*** **= 0.661**		**Agreement = 81.1%**						**3%**
			**NHANES diagnosis**						
**KNHANES diagnosis**			Normal		Osteopenia		Osteoporosis		
	*n*	%	*n*	%	*n*	%	*n*	%	
Normal	514	36.40	514	36.4	0	0	0	0	
Osteopenia	807	57.20	226	16.1	581	41.1	0	0	
Osteoporosis	91	6.40	0	0	40	2.8	51	3.6	
Total	1,412	100.00	740	52.5	621	43.9	51	3.6	
**70s**	***k*** **= 0.654**		**Agreement = 81.4%**						**6%**
			**NHANES diagnosis**						
**KNHANES diagnosis**			Normal		Osteopenia		Osteoporosis		
	*n*	%	*n*	%	*n*	%	*n*	%	
Normal	177	18.40	177	18.4	0	0	0	0	
Osteopenia	634	66.00	123	12.8	511	53.2	0	0	
Osteoporosis	150	15.60	0	0	56	5.8	94	9.8	
Total	961	100.00	300	31.2	567	59	94	9.8	
**80s**	***k*** **= 0.633**		**Agreement = 78.1%**						**9%**
			**NHANES diagnosis**						
**KNHANES diagnosis**			Normal		Osteopenia		Osteoporosis		
	*n*	%	*n*	%	*n*	%	*n*	%	
Normal	20	10.90	20	10.9	0	0	0	0	
Osteopenia	104	56.80	23	12.6	81	44.2	0	0	
Osteoporosis	59	32.30	0	0	17	9.3	42	23	
Total	183	100.00	43	23.5	98	53.5	42	23	

McNemar-Bowker *p*-value for all comparisons < 0.001.

NHANES, National Health and Nutrition Examination Survey; KNHANES, Korea National Health and Nutrition Examination Survey.

**Table 5:** **The change in diagnosis by the selected reference for females**

	***Cohen’s k***		**% of agreement**						**Prevalence difference**
**50s**	***k*** **= 0.457**		**Agreement = 69.5%**						**6%**
			**NHANES diagnosis**						
**KNHANES diagnosis**			Normal		Osteopenia		Osteoporosis		
	*n*	%	*n*	%	*n*	%	*n*	%	
**Normal**	741	53.10	390	28	351	25.1	0	0	
**Osteopenia**	622	44.60	0	0	547	39.2	75	5.4	
**Osteoporosis**	32	2.30	0	0	0	0	32	2.3	
**Total**	1,395	100.00	390	28	898	64.3	107	7.7	
**60s**	***k*** **= 0.446**		**Agreement = 71.1%**						**13%**
**KNHANES diagnosis**			**NHANES diagnosis**						
	Total		Normal		Osteopenia		Osteoporosis		
	*n*	%	*n*	%	*n*	%	*n*	%	
**Normal**	414	24.40	155	9.1	259	15.3	0	0	
**Osteopenia**	1,100	64.80	0	0	870	51.2	230	13.6	
**Osteoporosis**	183	10.80	0	0	0	0	183	10.8	
**Total**	1,697	100.00	155	9.1	1,129	66.5	413	24.4	
**70s**	***k*** **= 0.553**		**Agreement = 75%**						**19%**
**KNHANES diagnosis**			**NHANES diagnosis**						
	Total		Normal		Osteopenia		Osteoporosis		
	*n*	%	*n*	%	*n*	%	*n*	%	
**Normal**	101	7.90	27	2.1	74	5.8	0	0	
**Osteopenia**	742	57.90	0	0	496	38.7	246	19.2	
**Osteoporosis**	439	34.20	0	0	0	0	439	34.2	
**Total**	1,282	100.00	27	2.1	570	44.5	685	53.4	
**80s**	***k*** **= 0.565**		**Agreement = 82%**						**17%**
**KNHANES diagnosis**			**NHANES diagnosis**						
	Total		Normal		Osteopenia		Osteoporosis		
	*n*	%	*n*	%	*n*	%	*n*	%	
**Normal**	8	2.60	1	0.3	7	2.3	0	0	
**Osteopenia**	99	32.40	0	0	51	16.7	48	15.7	
**Osteoporosis**	199	65.00	0	0	0	0	199	65	
**Total**	306	100.00	1	0.3	58	19	247	80.7	

McNemar-Bowker *p*-value for all comparisons < 0.001.

NHANES, National Health and Nutrition Examination Survey; KNHANES, Korea National Health and Nutrition Examination Survey.

**Figure 2:**
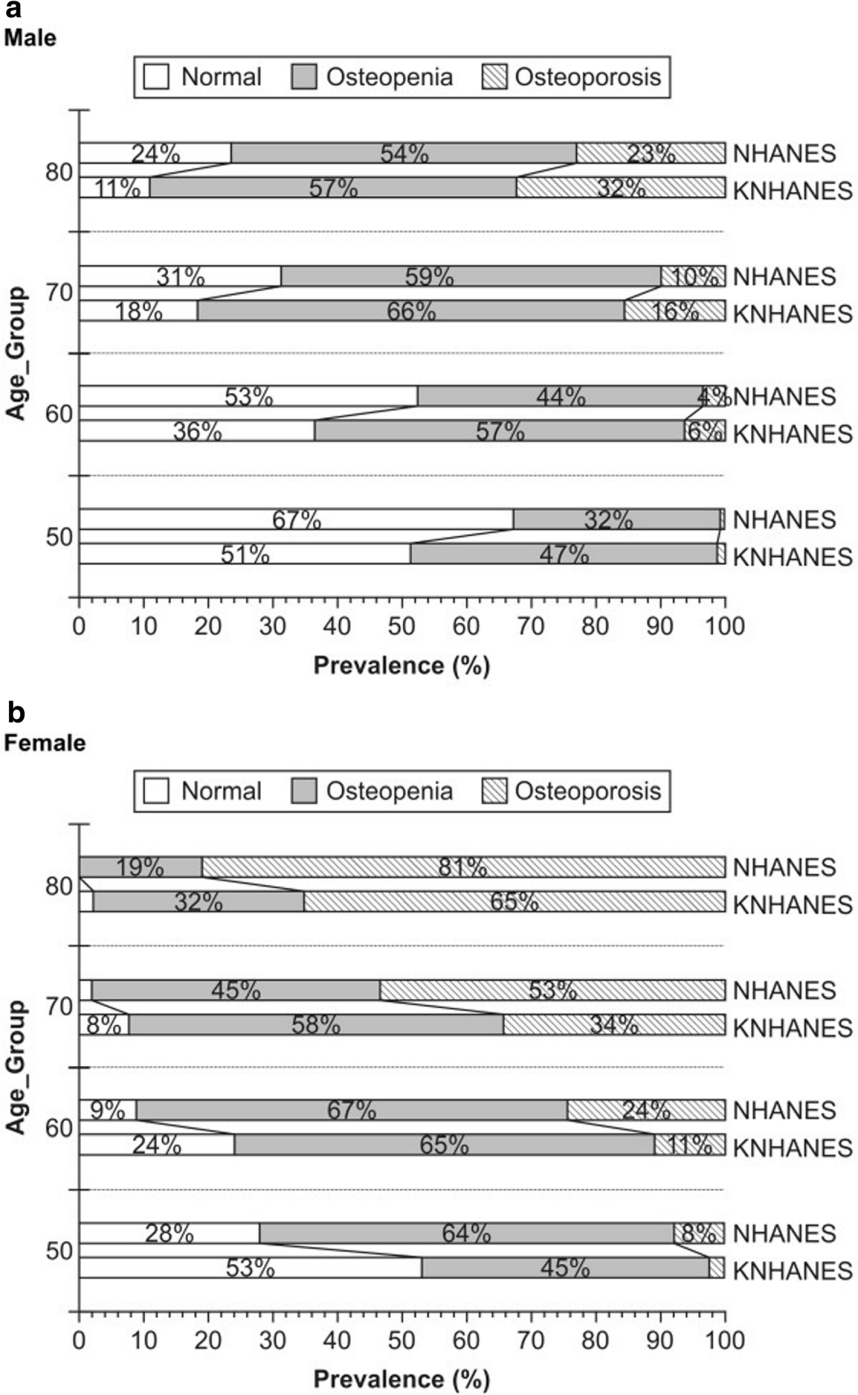
**The prevalence of osteoporosis and osteopenia by age group (a, male; b, female).** The differences in diagnoses from the reference are represented by sex and age groups. The gray portion and lined dark gray portion indicate the prevalence of osteopenia and osteoporosis, respectively. **(a)** In men, the prevalence obtained from the Korea National Health and Nutrition Examination Survey (KNHANES) male reference was higher than the values obtained from the NHANES III women reference across all age groups. **(b)** In men, the prevalence obtained from the NHANES III women reference was higher than the values obtained from the KNHANES female reference across all age groups.

Figure 2 compares the prevalence of osteoporosis and osteopenia according to the references. In men, when the Korean reference value was used, the prevalence of osteoporosis increased. In Figure 2 and Table 4, the difference in osteoporosis in men increased with age and reached 9% for those in their 80s. In women (Figure 2 and Table 5), the prevalence of osteoporosis was higher when the NHANES III white women reference was used. The differences in osteoporosis were 6%, 13%, 19%, and 17% for those in their 50s, 60s, 70s, and 80s, respectively.

### The difference in the fracture risk probability compared to the reference value

**Figure 3:**
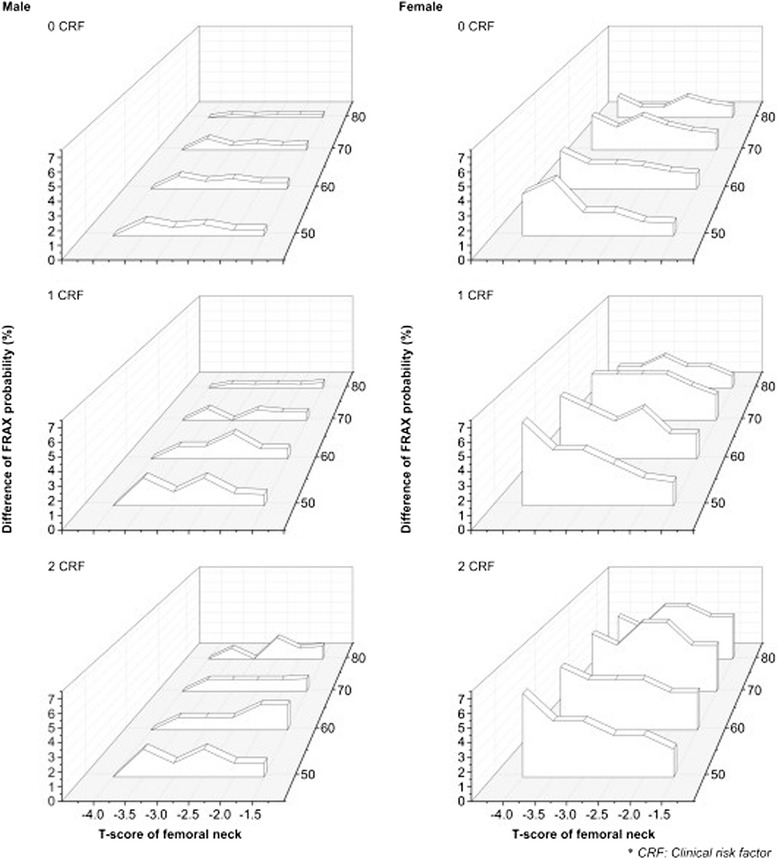
**Differences in the 10-year risk probability for major osteoporotic fractures by age and the number of clinical risk factors (CRFs).** The differences in the 10-year fracture risk probability for major osteoporotic fractures from the reference are shown for men in the left column and for women in the right column. Each row represents zero, one, or two CRFs in ascending order. The difference was higher in women with more clinical risk factors than in men. KNHANES, Korea National Health and Nutrition Examination Survey.

**Figure 4:**
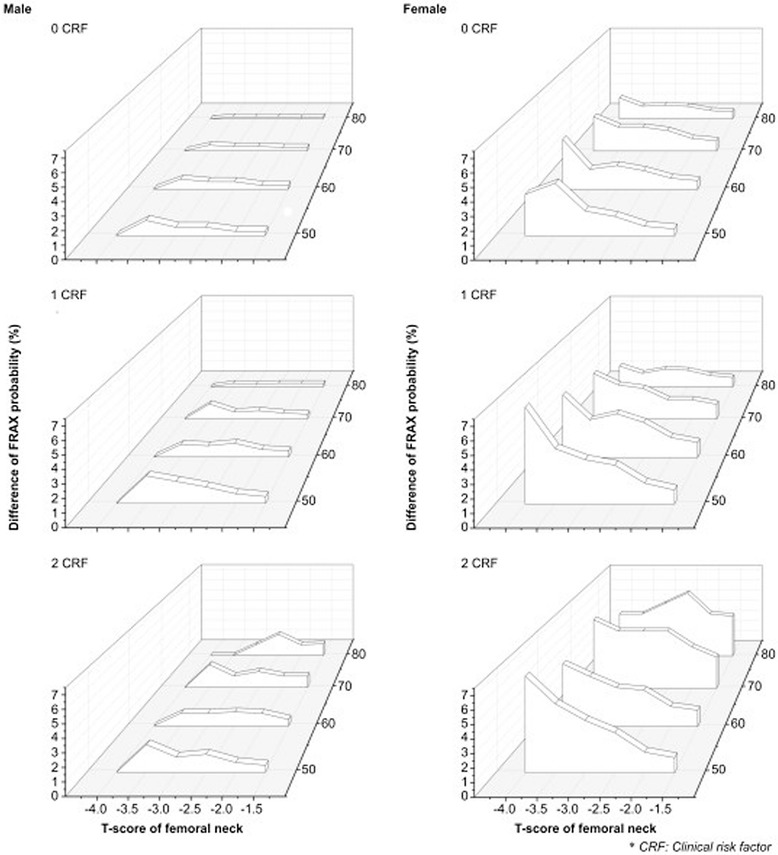
**Differences in the 10-year risk probability for femoral neck fractures by age and the number of clinical risk factors (CRFs).** The differences in the 10-year fracture risk probability for femoral fractures from the reference are shown for men in the left column and for women in the right column in. Each row represents zero, one, and two CRFs in ascending order. The difference was higher in women with more CRFs than in men.

Figure 3 and Table 6 show the difference in the 10-year major osteoporotic fracture probability. Figure 4 and Table 7 show the difference in the 10-year femoral neck fracture probability according to the T-score levels and age groups. The results are summarized as follows.

**Table 6:** **Difference in the estimated fracture risk probability for major osteoporotic fractures**

**Male**							
T-score		−1.5	−2	−2.5	−3	−3.5	−4
Korean Male		0.7119	0.6451	0.5783	0.5115	0.4447	0.3779
NHANES Female		0.6742	0.6147	0.5553	0.4958	0.4364	0.3769
**Age**	**No. of CRF**	**Male, Difference in the fracture risk probability (%)**					
50s	0	0.4	0.4	0.8	0.6	1	0
	1	0.8	0.9	2	1	2	0
	2	1	1	2	1	2	0
60s	0	0.5	0.5	0.8	0.6	1	0
	1	0.8	0.8	2	1	1	0
	2	2	2	1	1	1	0
70s	0	0.5	0.4	0.6	0.4	1	0
	1	0.7	0.7	0.9	0	1	0
	2	1.1	1	2	1	1	0
80s	0	0.3	0.3	0.3	0.2	0.3	0
	1	0.5	0.3	0.4	0.3	0.4	0
	2	1.2	1	2	0	1	0
**Female**							
T-score		−1.5	−2	−2.5	−3	−3.5	−4
Korean Female		0.6173	0.5650	0.5128	0.4605	0.4083	0.3560
NHANES Female		0.6742	0.6147	0.5553	0.4958	0.4364	0.3769
**Age**	**No. of CRF**	**Female, Difference in fracture risk probability (%)**					
50s	0	0.9	1	1.7	1.7	4	3
	1	1.7	2	3	4	4	6
	2	2	3	3	4	4	6
60s	0	1.2	1.4	1.8	2	2	3
	1	2	2	4	3	4	5
	2	3	3	4	4	4	5
70s	0	1.5	1.7	2.2	3	2	3
	1	2.2	3	4	4	4	4
	2	4	4	6	6	4	5
80s	0	1.1	1.4	2	1	1	2
	1	1.1	2	2	3	2	2
	2	4	4	5	5	3	4

KNHANES, Korea National Health and Nutrition Examination Survey; CRF, clinical risk factor.

**Table 7:** **Difference in the estimated fracture risk probability for femoral neck fractures**

**Male**							
T-score		−1.5	−2	−2.5	−3	−3.5	−4
Korean Male		0.7119	0.6451	0.5783	0.5115	0.4447	0.3779
NHANES Female		0.6742	0.6147	0.5553	0.4958	0.4364	0.3769
**Age**	**No. of CRF**	**Male, Difference in fracture risk probability (%)**					
50s	0	0.3	0.3	0.6	0.6	1.1	0
	1	0.5	0.7	1.2	1.6	2	0
	2	0.5	0.7	1.3	1.1	2	0
60s	0	0.3	0.3	0.6	0.6	0.8	0
	1	0.5	0.6	1.1	0.9	1	0
	2	0.5	1	1.1	1	1	0
70s	0	0.3	0.3	0.4	0.4	0.5	0
	1	0.4	0.5	0.7	0.6	1.3	0
	2	1	1	1.4	1	2	0
80s	0	0.2	0.1	0.2	0.2	0.2	0
	1	0.3	0.3	0.3	0.2	0.3	0
	2	1.1	1	2	1	0	0
**Female**							
T-score		−1.5	−2	−2.5	−3	−3.5	−4
Korean Female		0.6173	0.5650	0.5128	0.4605	0.4083	0.3560
NHANES Female		0.6742	0.6147	0.5553	0.4958	0.4364	0.3769
**Age**	**No. of CRF**	**Female, Difference in fracture risk probability (%)**					
50s	0	0.5	0.7	1.4	1.8	3.8	3
	1	1	1.5	2.8	3.2	4	7
	2	1.1	1.5	2.9	3.8	5	7
60s	0	0.7	0.9	1.5	1.9	1.6	4
	1	1.2	1.6	2.8	3.5	3	5
	2	1.3	1.6	2.9	3.1	4	5
70s	0	0.9	1.1	1.8	2.1	2.1	3
	1	1.3	1.7	1.7	2.7	3	4
	2	2.7	3.6	5	5	5	6
80s	0	0.7	0.8	1.2	1.3	1.2	2
	1	0.9	1.1	1.6	1.7	1.3	2
	2	3.8	4	6	5	4	4

KNHANES, Korea National Health and Nutrition Examination Survey; CRF, clinical risk factor.

Firstly, the differences in fracture risk were greater in women than in men. For both major osteoporotic fractures and femoral neck fractures, the difference of probability in men was <2%. However, the difference was higher in women. For major osteoporotic fractures, the highest difference identified was 6% for those in their 50s with a T-score of −4.0 and one or two clinical risk factors, and for those in their 70s with T-scores of −2.5 or −3.0 and two clinical risk factors. For femoral neck fractures, the highest difference was 7% for those in their 50s with a T-score of −4.0 and one or two clinical risk factors.

Secondly, the difference showed a tendency to increase as the number of clinical risk factors increased. For major osteoporotic fractures, this was most apparent in men aged 80–89 years with a T-score of −2.5; the difference of 0.3% with no clinical risk factor increased up to 1.7% with two clinical risk factors. In women aged 80–89 years, the difference of 1% with no clinical risk factor increased up to 5% with two clinical risk factors. For femoral neck fractures, the difference in men aged 80–89 years with no clinical risk factor increased from 0.2% to 2% with two clinical risk factors; for women in their 80s, the difference with no clinical risk factor increased from 1.2% to 6% with two clinical risk factors.

Finally, in women with a T-score ≤ −3.5 and none or one clinical risk factors, the difference in the major osteoporotic fracture risk decreased with age. However, in women with a T-score ≤ −3.0 and two clinical risk factors, the difference in the fracture risk increased with age. Table 6 shows data for women with a T-score −4.0 and one clinical risk factor, and the difference in the probability was 6%, 5%, 4%, and 2% for those in their 50s, 60s, 70s, and 80s, respectively. But the difference in women with T-score −2.5 and two risk factors increased from 3% to 5% in 2 risk factors with aging. The tendency was continued for femoral neck fracture in women with T-score ≥ −3.0 and two clinical risk factors, but the change in group with T ≤ −3.5 was slightly different little. The decreased tendency with aging can be seen in women with T-score between −2.5 to −4.0 and one clinical risk factor. Table 7 shows data for the femoral neck fractures in women with a T-score of −2 and two clinical risk factors, and the difference of probability increased from 1.5% for those in their 50s to 4% for those in their 80s. But, the difference in women with T-score −3.5 and one risk factor decreased from 4% to 1.3%.

## Discussion

This study showed a significant difference between the mean BMD of the NHANES III white women and of Korean males and females, which used the 20–29-year-old age group as a reference. The change in the reference value had a significant effect on the prevalence of osteoporosis and on the fracture risk probability. The difference in osteoporosis prevalence showed a rising tendency with age; the difference increased to 9% in men aged 80–89 years and to 19% in women aged 70–79 years. We indirectly investigated the difference in fracture risk probability using the FRAX™ probability; the difference in risk probability for major osteoporotic fractures from the change in the reference was up to 2% in men and 6% in women. In femoral neck fractures, the difference was up to 2% in men and 7% in women. The degree of difference was higher in women and in subjects with more clinical risk factors. For women with a T-score ≤ −3.5 and none or one clinical risk factor, the difference decreased with age. For women with a T-score ≥ −3.0 and two clinical risk factors, the difference increased with aging. This suggested that the change in the gradient of risk (the degree of fracture risk increased by 1 unit of decrease in the T-score) may have been caused by the change in the reference value.

Fracture risk assessment tools represented by the FRAX™ are being introduced quickly; however, a T-score ≤ −2.5 is still an important criterion for diagnosing and treating osteoporosis. The importance of a reference value was diluted by using the FRAX™, because it used the BMD value itself instead of a T-score. According to many guidelines, the T-score is still a criterion for diagnosing and treating osteoporosis [10,25,26]. In addition, there was controversy on the treatment duration of bisphosphonates, a therapeutic agent for osteoporosis. Most studies on this issue suggest the T-score as criteria for clinical decision making [27]. Considering the use of the T-score in real clinical situations, discussion relating to the selection of the reference value directly related to the T-score is still ongoing.

Currently, major guidelines such as the National Osteoporosis Foundation, International Society for Clinical Densitometry, WHO, and European guidelines recommend using the mean and SD of non-Hispanic white women aged 20–29 from the U.S. NHANES III BMD data as a reference and using the reference of women for osteoporosis diagnoses in women [9-11]. This recommendation was based on the fact that the difference of BMD by region was small compared with the difference in fracture incidence even if the BMD difference was significant [3]. However, the definition of small should be based on the effect of the reference on osteoporosis diagnoses and the assessment of fracture risk.

The differences of prevalence identified in this paper may have a significant effect on clinical decisions and health policies. In previous studies on Koreans, the prevalence of osteoporosis were reported as 0.3%, 2.6%, 7.9%, and 20.5% for men and as 5.3%, 16.8%, 43.4%, and 74.7% for women in their 50s, 60s, 70s, and 80s, respectively, when the manufacturer’s reference was used [28]. Our study showed that the differences in osteoporosis prevalence were 1%, 2%, 6%, and 9% for men and 6%, 13%, 19%, and 16% for women in their 50s, 60s, 70s, and 80s, respectively. These differences were considered significant compared with the aforementioned osteoporosis prevalence in Korea and can lead to under- or overestimation of the proportion of osteoporosis. Therefore, it may have an effect on the creation of health policies and the criteria of reimbursement, etc. Thus, as for the effect on clinical status, the differences from the change in the reference should not be determined as small, numerically speaking.

The prevalence of osteoporosis can have a significant effect on the cut-off value for the BMD test and treatment, which were the main elements in the guidelines for osteoporosis. Kanis et al. reported that the sensitivity and specificity of the fracture risk prediction changed with the proportion of the fracture risk group [29]. In this study, the proportion of saved fractures to the number screened also changed with the proportion of the fracture risk group. The prevalence of osteoporosis is a representative figure of the fracture risk group; thus, the change in the prevalence can affect the criteria for decision making during the BMD test and treatment. Therefore, the prevalence calculated by the Korean reference can provide more acceptable criteria.

We can consider the change in the T-score level for osteoporosis diagnosis (e.g., T-scores of −2.2 to −2.8 for osteoporosis diagnosis) with maintaining the NHANES III BMD data as the reference. By this methodology, the proportion of osteoporosis in a specific region can be held within a specific range [30]. Our study findings showed that the NHANES III women reference was higher than the KNHANES women reference; thus, the prevalence of osteoporosis increased using the NHANES III women reference. If the diagnostic criterion for osteoporosis increased to a T-score of −2.8, the prevalence may not increase even if NHANES III reference was used. For example, the osteoporosis prevalence diagnosed by the Korean reference of women aged 70–79 years was 34%. Assuming that this value was the real prevalence of osteoporosis in Korea, the prevalence can be consistent by using the criterion of a T-score of −2.8, even if the NHANES reference is used. However, by using this approach, the definition for diagnosing osteoporosis changes from a T-score of ≤ −2.5 to a T-score of ≤ −2.8, which may cause confusion among health care providers and health policy makers, causing a significant increase in the cost of time and money. In Korea, it took more than 5 years to change the insurance standards for diagnosing osteoporosis with a T score of ≤ −3.0 to a T-score of ≤ −2.5.

A reason for using the NHANES III data was that there were few well-structured BMD databases in many areas [12,13]. The KNHANES 2008–2011 database was equivalent to the NHANES III non-Hispanic white women database in terms of sample size and study design. The KNHANES 2008–2011 reference group was composed of 976 males and 1,236 females aged 20–29 years compared with 409 women of the NHANES III non-Hispanic white women reference data. It was designed well with an adequate sampling method and study design. In addition, the Korean BMD reference data better reflected the characteristics of the Korean BMD.

The change caused by selecting references can affect the relationship between the T-score and the prediction of fracture risk. In a 60-year-old woman with two clinical risk factors (i.e., a previous fracture history and family history for fracture), the difference in the 10-year risk probability for femoral neck fractures, according to the Korean version of the FRAX™, was 2.9% between the Korean women reference and the NHANES III women reference. The difference in a 70-year-old woman and an 80-year-old woman in the same conditions was 5% and 6%, respectively. This difference was decreased to 1.5% for those in their 60s, 1.8% for those in their 70s, and 1.2% for those in their 80s. According to findings in previous studies, the 10-year risk probability for hip fracture was <5% for those in their 60s, 9% for those in their 70s, and 13% for those in their 80s [29]. When comparing our study findings to those of previous findings, the size in difference from each reference was significantly large.

The difference in the BMD from the same T-score was according to the range of subject’s T-score. In Tables 5 and 6, the difference in the BMD from the same T-score decreased with a decreasing T-score. In men, the BMD differences were 0.0377 g/cm^2^, 0.0304 g/cm^2^, 0.023 g/cm^2^, 0.0157 g/cm^2^, 0.0083 g/cm^2^, and 0.001 g/cm^2^ for T-scores of −1.5, −2.0, −2.5, −3.0, −3.5, −4.0, respectively. The difference in women was greater than men. And, the BMD differences also decreased with a decreasing T-score (0.0569 g/cm^2^, 0.0497 g/cm^2^, 0.0425 g/cm^2^, 0.0353 g/cm^2^, 0.0281 g/cm^2^, and 0.0209 g/cm^2^ for T-scores of −1.5, −2.0, −2.5, −3.0, −3.5, and −4.0, respectively). As a result, the difference in the fracture risk probability was according to sex and T-score. The difference in the BMD from the same T-score was larger in women whose T-score was > −3.5. This concluded that the reference change had a significant effect on women with approximate T-score of −2.5, for whom the diagnosis and decision making of treatment was changed sensitively.

Our study has some limitations. First, it did not provide the degree of distribution of risk factors in the groups. In the Korean version of the FRAX™, the risk factors that had a significant effect on the fracture risk probability were a history of fracture, family history of fracture, and a history of steroid use. KNHANES 2008–2011 did not have information on family history or a history of steroid use, but it included the data on a history of previous fracture (*n* = 353, 8.6%). Second, instead of the real fracture risk incidence, we used the estimated fracture risk probability from the FRAX™. Ideally, the relationship between the fracture incidence rate and the change in reference was analyzed by regression analysis with an adjustment for age, the number of clinical risk factors, and the T-score. Because of these limitations, we presented only a trend according to the associated factors, and we were unable to provide statistical significance for them as well as a better explanation for their tendency.

## Conclusions

The reference value has a significant effect on the prevalence of osteoporosis, and it affects the function of the BMD for predicting the incidence of fractures. When this significance is considered, the BMD data of the KNHANES 2008–2011 reflected the characteristics of the Korean population more precisely; therefore, it can provide more realistic criteria for diagnosing and treating osteoporosis in Korea.

## Abbreviations

KNHANES: Korea National Health and Nutrition Examination Survey
NHANES: National Health and Nutrition Examination Survey
FRAX: Fracture risk assessment tool
DXA: Dual energy radiography absorptiometry
BMD: Bone mineral density
WHO: World Health Organization
